# Thyroid surgery during the COVID-19 pandemic: difficulties – how to improve

**DOI:** 10.1515/iss-2022-0015

**Published:** 2022-10-11

**Authors:** Christos K. Stefanou, Georgios Papathanakos, Stefanos K. Stefanou, Kostas Tepelenis, Aikaterini Kitsouli, Alexandra Barbouti, Stefanos Flindris, Periklis Tsoumanis, Panagiotis Kanavaros, Panagiotis Kitsoulis

**Affiliations:** Department of Surgery, General Hospital of Filiates, Thesprotia, Greece; Intensive Care Unit, University Hospital of Ioannina, Ioannina, Greece; Department of Endocrine Surgery, Henry Dunant Hospital Center, Athens, Greece; Department of Surgery, University Hospital of Ioannina, Ioannina, Greece; Department of Anatomy-Histology-Embryology, University of Ioannina, Ioannina, Greece; Department of Obstetrics and Gynecology, University Hospital of Ioannina, Greece; Department of Ophthalmology, University Hospital of Ioannina, Ioannina, Greece; Department of Orthopaedics, University of Ioannina, Ioannina, Greece; Medical School, University of Ioannina, Ioannina, Greece

**Keywords:** Covid-19 pandemic, fine needle aspiration cytology, preoperative diagnosis, thyroid surgery, translaryngeal ultrasonography

## Abstract

In December 2019, the new coronavirus infection (COVID-19) was declared a pandemic by the World Health Organization after rapidly spreading over the world in just a few months. All elective operations and nonemergency treatments have been postponed worldwide. However, some patients require surgical therapy as well, and the time spent waiting should not have a negative impact on the surgical outcome or disease course. Following the initial onset of the COVID-19 epidemic, instructions for proper and safe surgery for healthcare staff and patients should develop. Thyroid surgeries have decreased during the COVID-19 pandemic. Most of them can be postponed for a long time. Assessment of thyroid nodules recommends clinical examination, imaging studies, fine needle aspiration (FNA) and vocal cord examination. All these procedures are necessary, and sometimes they cannot be postponed. To determine the best timing, a thorough preoperative assessment should be undertaken, taking into account both oncological and anatomical features. Furthermore, COVID-19 status must be negative prior to any intervention, and hospital infrastructure must be ready to deal with the demanding situation.

## Introduction

SARS coronavirus 2 (SARS-CoV-2) caused Coronavirus Disease 2019 (COVID-19), which was initially detected in Wuhan, China, in December 2019 and declared a pandemic by WHO on March 11, 2020 [1].

Governments worldwide have undertaken social distance, lockdowns, quarantines, travel restrictions, and hygiene measures to slow the spread of the infection. The quality of care for numerous illnesses has changed due to reallocating resources to treat COVID-19 patients, resulting in hospital beds and staff shortages and prioritizing cases to decrease transmission and relieve healthcare systems [2]. The ongoing COVID-19 pandemic commands a significant reorganization of the healthcare system.

Several cancer patients have been advised to limit their contact with medical facilities to minimize nosocomial infections. As a result, a significant number of cancer patients (38.7–59.0%) have been reported to have experienced a COVID-19-induced therapy delay [3, 4]. Thyroid carcinoma is the most common endocrine system cancer, with a threefold increase in incidence over the last two decades [5].

The risk of contamination to patients and healthcare workers who are COVID-19 negative, hospital facilities, especially the conditions related to the preoperative management, the operating room and postoperative care, should be considered when planning surgery during and after the COVID-19 pandemic [6].

Most thyroid surgeries can be postponed for a long time. However, it must be remembered that those patients require surgical treatment as well and that the time spent waiting should not produce delays that could affect the surgical outcome or disease progression [6].

This review aims to evaluate the impact of the COVID-19 pandemic on thyroid surgery. Especially the difficulties and how we can improve thyroid surgery during the demanding situation of this pandemic.

## Difficulties

### Delayed diagnosis

Two of the essential protection against the transmission of COVID-19 are social distancing and social isolation [7]. These protections resulted in changes to the medical system and the medical approach to the patients with fewer medical assessments.

Tsang et al. found that telehealth (video consultations) became a strategy to assess patients, and 80% of patients transferred from face to face consultations to virtual ones in May 2020 [8].

Despite patient and physician satisfaction with telemedicine, physical examination and ultrasound were impossible, and the assessment was poor.

The diagnostic approach to thyroid nodules consists of ultrasound, scintigraphy, FNA and changed during the pandemic. Less often, FNAs were conducted during this period, and more samples were malignant. Palladino et al. analyzed the number of FNAs before the pandemic (January 2019–13 March 2020) and during the pandemic (14 May 2020–7 July 2020) [9]. They found that the number of weekly FNAs dropped from 62.1% to 23.1%, and the high-risk diagnoses from these samples increased by 6% [9].

Zhang et al. found that during the pandemic and the phase I (highest alert), FNAs were conducted 99.7% less often than FNAs before the pandemic. During the pandemic phase II and III FNAs trends were 30.1% lower than before the pandemic, and approximately half of the samples were malignant [10].

Ultrasound of the thyroid gland is a noninvasive examination and the first step in evaluating the thyroid parenchyma and its nodules [11]. The noninvasive nature of this examination makes it easy and quick to use, with low cost, but it is an operator-dependent examination. The thyroid nodule grading system that has been proposed is TIRADS. There are the following nodule classification systems, EU-TIRADS, K-TIRADS, ACR-TIRADS and BTA-TIRADS. EU TIRADS had the highest sensitivity (82.7%); ATA had the highest specificity (66.4%). The study by Dutta et al. showed that FNA and the TIRADS system showed that both systems have a sensitivity of 80%. Ultrasound findings show microcalcifications have a sensitivity of 80% and a specificity of 86%. Also, the irregular shape and the taller than wider parameter have a sensitivity of 89 and 92%, respectively [12]. During the pandemic, less contact with the patient is the first choice of the doctor and the health systems. The development of remote ultrasound is a safe option, although it is still at an early stage. The study by Jiang W et al., on 22 patients with COVID-19 infection showed that it is possible to remotely perform an ultrasound examination using robotic arms and the 5 g network [13].

Since only around 10% of all thyroid nodules will be cancerous, it has been acceptable to postpone most thyroid biopsies during the pandemic, except those nodules with extremely worrying sonographic signs according to the TIRADS scoring system or clinical indications of blockage [14]. According to Liu et al., this strategy resulted in more aggressive thyroid cancer. The patients were more likely to have multiple sessions (31.2% before COVID-19 vs. 36.5% during covid19), extrathyroidal extension (65.5% vs. 72.2%) and lymph node metastases (37 vs. 45%) while the size of the tumor remained stable (1.01 vs. 1.02) [15].

### Decrease in thyroidectomies

During the pandemic, the number of elective surgeries decreased due to the risk of infection in patients and surgeons and also to allow a better allocation of resources. Since thyroid surgery is mostly not an emergency, all patients who require thyroid surgery experience delays during the pandemic [16].

For surgery patients, COVID-19 infection increases the risk of pre- and post-operative complications. Even in many high-income countries, the surgeries are decreased and limited to oncological and emergency surgeries [17].

Medas et al., analysed in a multicentric, retrospective study with 3,892 patients the impact of the pandemic on thyroid surgery. The number of operations decreased by 64.8% (1st phase), 44.7% (2nd phase) and 5.1% (3rd phase) during the COVID 19 pandemic compared to 2019 [18]. In comparison to the same period the previous year, they discovered that the incidence of cancers has more aggressive characteristics [18].

### Post-operative complications

Wai et al. and Lombardi et al., showed that the number of patients with postoperative complications during the pandemic was not significantly different compared to the situation before [19, 20]. On the other hand, Zhang et al., observed a significant increase in vocal cord paralysis. The mean postoperative hospital stay was reduced by 0.4 days compared with 2019 [20].

A multicentric, international (26 countries) observational study for head and neck surgery (thyroid cancer was 21% of the cases) showed a 1.2% 30-day mortality, 3% of covid-19 positive at the first month of the operation. The infection was more severe in patients with a more advanced tumor [21].

Scappaticcio et al., collected data from thyroidectomies during the pandemic. Cross infections had the 1.9% of cases, and 0.4% had severe pulmonary complications of COVID-19. As far as other complications are concerned, 75.5% developed hypoparathyroidism and 18.8% recurrent laryngeal nerve injury [22].

Studies on whether hormonal dysfunctions or complications from thyroidectomy affect the clinical course of COVID-19 infection are lacking.

## How to improve?

### FNA

FNA is a cost-effective and highly sensitive approach for determining the nature of thyroid nodules. The role of FNA is essential in the diagnostic pathway and the main reason for his routine use.

The selection of FNA patients during the pandemic has become an issue. In reality, performing FNA indiscriminately in a population with a high prevalence of thyroid nodules may result in a low cost-benefit ratio, resulting in lower diagnostic efficiency and a greater frequency of inadequate/unsatisfactory FNA results [23]. Palladino et al., showed that the total number of weekly FNAs reduced from 62.1 to 23.1% during the pandemic. The weekly proportion of benign diagnoses decreased by 12%, while the high-risk proportion increased by 12%. It is safe if you prioritize the FNA procedure only for the high-risk thyroid nodules patient [9].

The production of the slides is usually air-dried with Romanowsky stain (Diff-Quik, May-Grünwald-Giemsa), a quick and useful way to improve pleomorphism and distinguish extracellular from intracytoplasmic material, allowing good definition of the cell outline and cytoplasmic contents. On the other hand, alcohol-fixed slides with Papanicolaou (Pap) stain allow clearer visualization of cellular morphology and nuclear features [24].

The slide preparation could be dangerous during the pandemic due to the potentially infectious material. Liquid-based cytology (LBC) methods in thyroid FNA can reduce the risk of aerosol diffusion of potentially infected material. Rossi et al. showed that the LBC method provided similar diagnostic results to the conventional method and can be applied during a pandemic and provide more safety. No significant differences between cytological and histological diagnoses were noticed in patients with potentially malignant lesions [25].

### Preoperative assessment

All patients should be regarded as suspicious during the COVID-19 pandemic, and illness and infection control measures should be performed. The COVID-19 status of the patient should be assessed before starting with any invasive therapeutic technique or surgical intervention. The gold standard assessment method is a nasopharyngeal swab sample followed by a reverse transcription PCR assay. If the patient tests negative and the clinical suspicion is still there, the test should be repeated for the next 24–48 h [26].

According to the Association of Anaesthetics, the recommendation for patients with elective surgery should be individualized risk assessment and be planned within 7 weeks of SARS-CoV-2 infection [27]. If surgery is considered, the physician should discuss the risks and benefits:

a) The risk of mortality calculated using a validated risk score
b) Risk modifiers based on patient factors, SARS-CoV-2 infection (timing, symptoms) and surgical factors (risk of disease, the complexity of surgery)

Within 10 days of a diagnosis of SARS-CoV2 infection, patients should not undergo elective surgery because they may be contagious and pose a risk to the staff and other patients [27].

Even after 7 weeks, patients with ongoing symptoms and those with moderate to severe COVID19 (such as those who were hospitalized) are still likely to be at higher risk of morbidity and mortality. As a result, postponing surgery, weighing this risk against any risks connected to the delay [27].

Whenever possible, preoperative COVID-19 vaccination should be given to surgical patients in three doses, with the last dose given at least two weeks before the surgery [27].

Clinical examination and blood tests are suggested for all patients as the preoperative assessment regardless of the COVID 19 screening status.

### Surgical triage

#### Thyroid cancers

After the COVID-19 pandemic, the selection of cancer patients for surgical management has changed due to the significant risk of infection among patients and health professionals. Clinical and histological features are the most widely used criteria for proper patient selection.

Acute airway compression is an urgent surgery indication, which should be performed even in the early pandemic phases, when the pressure on hospitals is greatest [28]. Patients with locally advanced tumors with invasion of surrounding vital structures (e.g., recurrent laryngeal nerve, trachea, esophagus, major vascular structures) and large, compressive or fast-growing differentiated tumors with concurrent nodal involvement should be considered as candidates for surgery. Anaplastic poorly differentiated and medullary thyroid tumors are the most common histological types requiring immediate surgery [28].

Surgical treatment for most well-differentiated carcinomas, often slow-growing and have an excellent prognosis, will depend on lymph node and tumor size. Tumors ≥2 cm with or without lymph nodes can be postponed until the pandemic is finished without risk, but they must be given priority and scheduled within the next three months. Tumors <2 cm without lymph node metastases can be postponed till after the pandemic is over [29].

#### Benign thyroid disorders

For most benign thyroid disorders thyroidectomies can be postponed without risk. Thyrotoxicosis (Graves’ disease, toxic nodules, toxic goiters, iatrogenic hyperthyroidism) resistant to or poorly controlled by synthetic anti-thyroid (SAT) medicines, for example, may necessitate semi-urgent scheduling [28]. Nonsuspicious goiters causing significant compressive symptoms (inspiratory dyspnea from tracheal compression, dysphagia from esophageal compression, superior vena cava syndrome from deep vein compression) must be scheduled for semi-urgent surgery before the pandemic ends [28].

### Vocal cord examination

Although clinical voice evaluation is required before thyroid surgery, indirect laryngoscopy (IDL), when necessary, has been questioned due to the possibility of aerosolized SARS-CoV-2 during endoscopy. Aygun et al. recommend that endoscopic vocal cord examination should not be done regularly but only in selected cases of hoarseness with the proper personal protection equipment during the test. Only patients with recurrent laryngeal nerve injury or loss of signal during intraoperative neuromonitoring should have a laryngeal exam during the postoperative period or if further contralateral surgery is anticipated soon [29].

**Figure 1:**
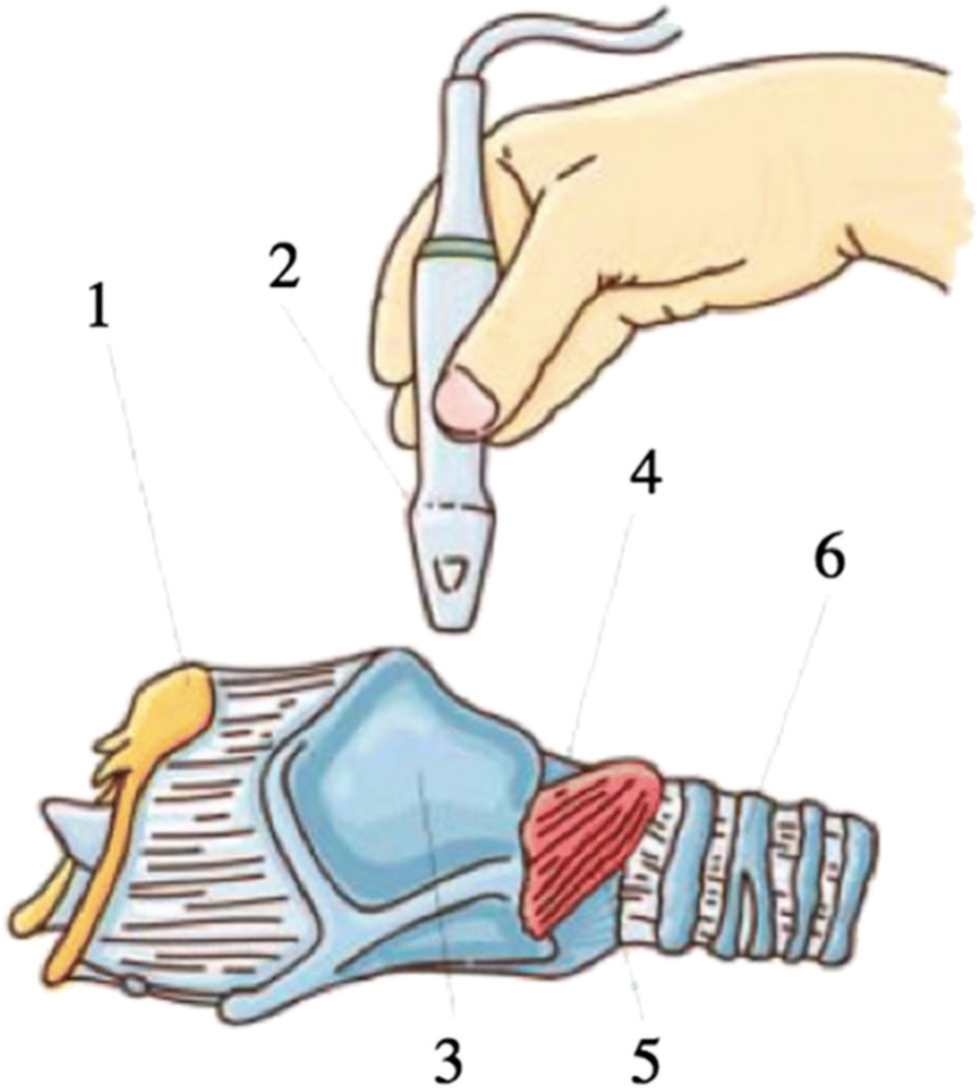
Placement of the ultrasound transducer on the neck. (1) Hyoid bone, (2) ultrasound transducer, (3) thyroid cartilage, (4) cricoid cartilage, (5) cricothyroid muscle, and (6) trachea.

**Figure 2:**
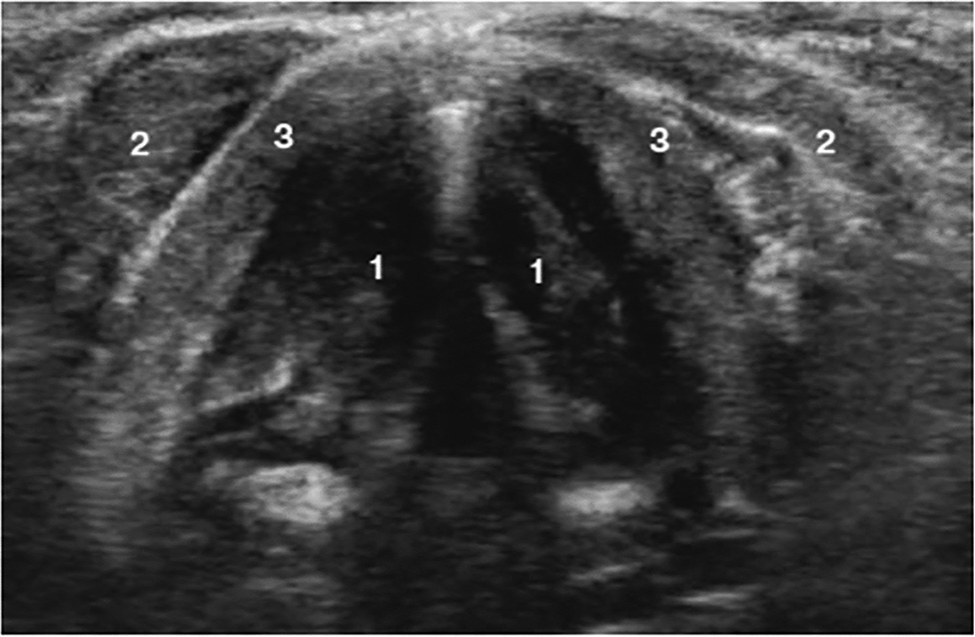
TLUS of the vocal cords in the open (quiet) position in the B-scan. (1) The true vocal cords, (2) short muscles of the neck, and (3) thyroid cartilage.

**Figure 3:**
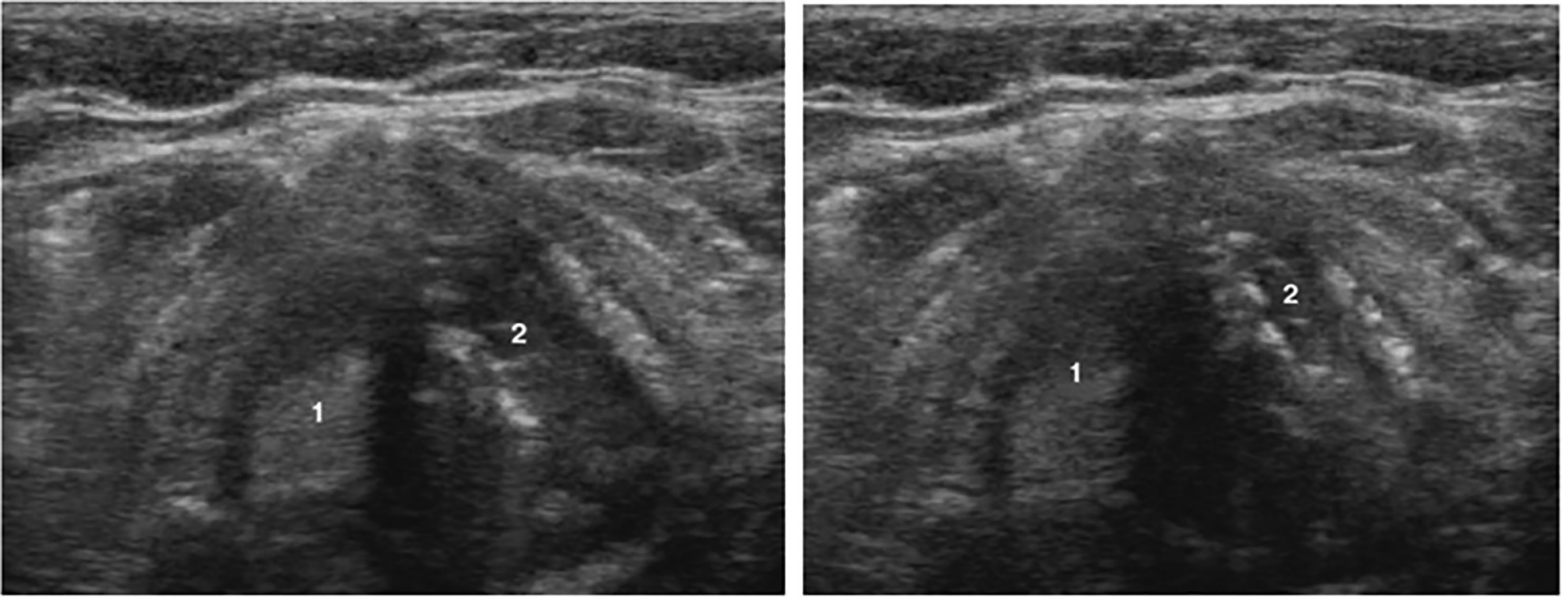
TLUS of the of right vocal cord palsy. (1) Right vocal cord is static and (2) left vocal cord is moving.

To examine the vocal cords, translaryngeal ultrasonography (TLUS) has been offered as a safe, noninvasive, and sensitive alternative. The patient is placed in a flat position with his neck slightly extended of the neck during TLUS. The ultrasound transducer is placed transversely over the anterior aspect of the middle portion of the thyroid cartilage (Figure 1). False cords and true cords were identified using sonographic landmarks whenever possible. The true VCs are the hypoechogenic structures projected under the thyroid cartilage (Figure 2). Unilateral palsy in B-scan could be visualized as immobility of the VC during phonation or Valsalva maneuver (Figure 3).

According to Knyazeva et al., patients who are female and under 50 years old are more likely to have their vocal cords visualized by TLUS (89% of patients visualize their vocal cords) [30]. According to structural features (a more acute angle of the thyroid cartilage) and laryngeal calcification, the approach does not appear accurate for male patients. It has been suggested that TLUS is a commonly used, noninvasive treatment [30]. It is convenient for the patient and does not increase the financial cost of preoperative workup because the thyroid surgeon himself can perform it. Furthermore, the mean length of the ultrasound examination time is significantly shorter than the time spent performing flexible laryngoscopy (1.8 ± 0.86 min vs. 3.4 ± 1.06 min; p<0.0001) as reported by Masood et al. [31] TLUS is an effective method for function control of the vocal cords in most patients undergoing thyroid and parathyroid surgery. The vast majority of surgery candidates, who are typically female and young, might benefit from using this visualizing technique. Laryngeal calcifications are a limiting concern, necessitating the use of the DFL in 20% of cases, particularly in male patients [30].

Thus, TLUS is an alternative to IDL in the post-operative scenario especially in the young female patient with a normal voice on clinical examination, to establish recurrent laryngeal nerve integrity while reducing the risk of aerosolization [32].

### Operation room

Hospitals should be prepared and organized to perform urgent surgical procedures, particularly for infected patients, to save the patient and healthcare workers from infection and prevent the hospital environment from being contaminated, thereby saving other patients and hospital staff [33]. If possible, suspect or confirmed COVID-19 cases should be scheduled at the end of the list. The patient should be transferred with a surgical mask.

The move to the operation room must take place as soon as possible, with minimum contact with the hospital environment and other people. If necessary, a COVID-19 positive patient elevator should be employed, and urgent disinfection should be conducted after the transfer. Infected patients must have their own operating room, apart from the rest of the operating complex. Negative pressure operation rooms are necessary to prevent contamination in the halls and other areas of the operational complexity.

Before the patient is admitted to the OR, all equipment and surgical objects are prepared in the room. COVID-19-infected patient’s surgeries should be arranged to be completed as rapidly as feasible with the fewest possible surgical personnel. The surgical team in charge during the procedure should be determined ahead of time, recorded, and allowed inside the room [34].

### Follow up

Hypocalcemia is a possible postoperative complication and Aygun et al., recommend that these patients should be treated as outpatients. By contrast, patients with severe hypocalcemia should be treated in the hospital until it is confirmed that discharge bears only a low risk of readmission [29].

Aygun et al., propose treating patients with levothyroxine until additional testing is available because thyroid function tests may be difficult to get because of the pandemic [29].

Baud et al. advise teleconsultation for longer-term follow-up to ensure continuity of care while lowering the danger of SARS coronavirus transmissions. According to the study, primary care clinicians should also undertake follow-up blood and imaging tests [35].

## Conclusions

Studies and systematic reviews should follow accepted methodological guidelines during public health emergencies to give patients, doctors, and decision-makers reliable information. The disease itself, social distancing, and the devastating economic impact are just a few of the factors that play a significant part in the pandemic. COVID-19 spreads at an alarming rate all over the world and has caused extraordinary societal instability, resulting in a rapid and still-ongoing global restructuring of health systems. During the pandemic, both diagnostic and therapeutic surgical procedures for thyroid cancer have decreased. The indolent nature of many thyroid tumors may allow for postponed treatment, but patients should be informed of this subtlety. Although active surveillance for a predetermined amount of time can be a safe alternative for low-risk patients, anaplastic, weakly differentiated, medullary, and advanced differentiated thyroid cancer management should not be postponed.

**Table:** 

Summary		
**Diagnosis**	– Decrease of diagnostic examinations – FNA-LBC can reduce the risk of transmission – TLUS is a safe and noninvasive examination for vocal cord evaluation	
**Surgical triage**	Thyroid cancer – Locally advanced tumors with invasion of surrounding vital structures – Anaplastic poorly differentiated and medullary thyroid tumors need immediate surgery – Well differentiated carcinomas can be postponed from 3–6 months	Benign thyroid Disorders – Resistant thyrotoxicosis necessitate semi urgent surgery – Nonsuspicious goiters causing compressive symptoms need semi urgent surgery
**Preoperative management**	– COVID 19 status screening – Elective surgery should be individualized risk assessment and be planned within seven weeks of SARS-CoV-2 infection. – Personal protective equipment – Specially designed operating rooms (negative pressure etc.)	
**Follow up**	– Telemedicine is a safe option for long term follow up – Hypocalcemia can be treated as an outpatient	

## Supplementary Material

Supplementary Material
