# Supplementary material for: Thyroid surgery during the COVID-19 pandemic: difficulties – how to improve
Source: Innov Surg Sci. 2022 Oct 11;7(3-4):125–32. doi: 10.1515/iss-2022-0015 (PMC9742267; doi:10.1515/iss-2022-0015)
Supplement: Supplementary file 1 — Supplementary Material [file j_iss-2022-0015_suppl.pdf]

## Reviewer Assessment

# C. Stefanou et al.: Thyroid surgery during the COVID 19 pandemic: Difficulties - How to improve

### Reviewers' Comments to Original Submission

**Reviewer 1: anonymous**

Date received: 28-Jul-2022
Reviewer recommendation: **Return to author for major modifications**
Reviewer overall scoring: **Medium**

Assessment Form scores: 5 = High/Yes; 3 = Medium/Adequate; 1 = Low

| | | | | | |
|---|---|---|---|---|---|
| Is the subject area appropriate for the journal | | | 3 | | |
| Does the title clearly reflect the paper's content? | | 4 | | | |
| Does the abstract clearly reflect the paper's content | | 4 | | | |
| Do the keywords clearly reflect the paper's content? | | | | 2 | |
| Does the introduction present the problem clearly? | | | 3 | | |
| Are the results/ conclusions justified? | | | 3 | | |
| How comprehensive and up-to-date is the subject matter presented? | | 4 | | | |
| How adequate is the data presentation? | | 4 | | | |
| Are units and terminology used correctly? | | | | 2 | |
| Is the number of cases adequate? | | | | 2 | |
| Are the experimental methods/ clinical studies adequate? | | | | 2 | |
| Is the length appropriate in relation to the content? | | | 3 | | |
| Does the reader get new insights from the article? | | | 3 | | |
| Please rate the practical significance. | | 4 | | | |
| Please rate the accuracy of methods. | | | 3 | | |
| Please rate the statistical evaluation and quality control. | | | | 2 | |
| Please rate the appropriateness of the figures and tables. | | | 3 | | |
| Please rate the appropriateness of the references. | | 4 | | | |
| Please evaluate the writing style and use of language. | | 4 | | | |
| Please judge the overall scientific quality of the manuscript. | | | 3 | | |
| Are the methods used worthy of reproduction in greater deal? | No | | | | |
| Would you be willing to review a revision of this manuscript? | yes | | | | |

**Comments to author:** Dear Authors,
thank you very much for giving me the chance to review the manuscript.
The article is of interest due to the ongoing pandemic situation. The challenges described herein are still of clinical relevance. I have to admit that the suggestions how to manage patients with thyroid disease during the pandemic is not completely new

to me, but I am an endocrine surgeon so that I am very familiar with these patients. For the general surgeon, however, the paper gives relevant Information. For this reason, I can recommend publication of the manuscript after major revision:
1. I suggest a change of key words: Covid-19 Pandemic, Thyroid Surgery, Preoperative Diagnosis, Fine Needle Aspiration Cytology, Translaryngal Ultrasonography
2. I suggest that the authors present some figures of TLUS. They also should describe in more detail the chances and challenges when applying this method of examination of the vocal cords.
3. When should a patient after a Covid-19 infection be operated on? What do the authors recommend in case of an infection although vaccinated?
4. Are there data that the materail obtained by FNA is contaminated by the Sars-Cov2-Virus? If so, please describe.
5. What is the relevance of preoperative ultrasound? I miss data about that, in particular with respect to TRAS-classification: Please describe.
One again, thank you for giving me the chance to review. I am looking forward to re-evaluate the revised manuscript.

**Reviewer 2: Geffcken, Christine**

Date received: 28-Jul-2022
Reviewer recommendation: **Accept in present form**
Reviewer overall scoring: **High**

Assessment Form scores: 5 = High/Yes; 3 = Medium/Adequate; 1 = Low

| | | | | | |
|---|---|---|---|---|---|
| Is the subject area appropriate for the journal | 5 | | | | |
| Does the title clearly reflect the paper's content? | 5 | | | | |
| Does the abstract clearly reflect the paper's content | 5 | | | | |
| Do the keywords clearly reflect the paper's content? | 5 | | | | |
| Does the introduction present the problem clearly? | 5 | | | | |
| Are the results/ conclusions justified? | | | 3 | | |
| How comprehensive and up-to-date is the subject matter presented? | | 4 | | | |
| How adequate is the data presentation? | 5 | | | | |
| Are units and terminology used correctly? | 5 | | | | |
| Is the number of cases adequate? | 5 | | | | |
| Are the experimental methods/ clinical studies adequate? | | 4 | | | |
| Is the length appropriate in relation to the content? | | 4 | | | |
| Does the reader get new insights from the article? | | | 3 | | |
| Please rate the practical significance. | | | 3 | | |
| Please rate the accuracy of methods. | | 4 | | | |
| Please rate the statistical evaluation and quality control. | 5 | | | | |
| Please rate the appropriateness of the figures and tables. | 5 | | | | |
| Please rate the appropriateness of the references. | 5 | | | | |
| Please evaluate the writing style and use of language. | 5 | | | | |
| Please judge the overall scientific quality of the manuscript. | | 4 | | | |
| Are the methods used worthy of reproduction in greater deal? | Yes | | | | |
| Would you be willing to review a revision of this manuscript? | Yes | | | | |

**Comments to author:** good article, no major critical points.

I recommend a clear conclusion, that the role of the pandemic is multifactorial (disease itself, social distancing, and other resulting effects - it is not proved which effects are the most).

## Authors' Response to Reviewer Comments

Date received: 15-Aug-2022

### Response to reviewer 1

1) *Key words. Covid-19 Pandemic, Thyroid Surgery, Preoperative Diagnosis, Fine Needle Aspiration Cytology, Translaryngeal Ultrasonography*

2) *Figures from TLUS:*

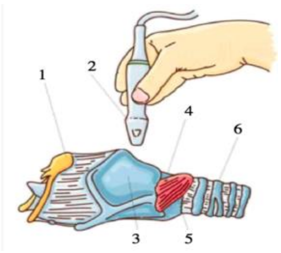


Figure 1. Placement of the ultrasound transducer on the neck. (1) Hyoid bone,

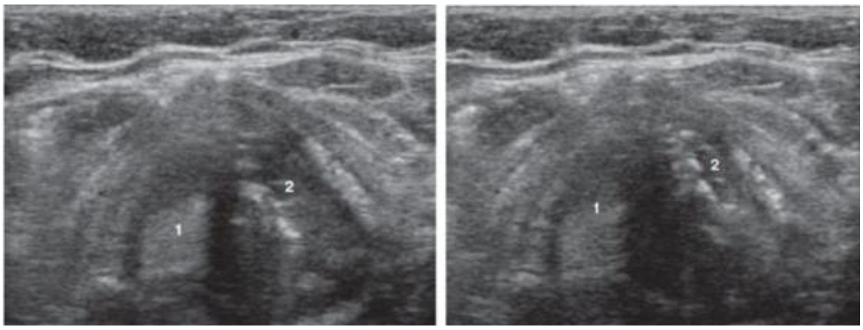


Figure 3. TLUS of the of right vocal cord palsy. (1) Right vocal cord is static and (2) left vocal cord is moving

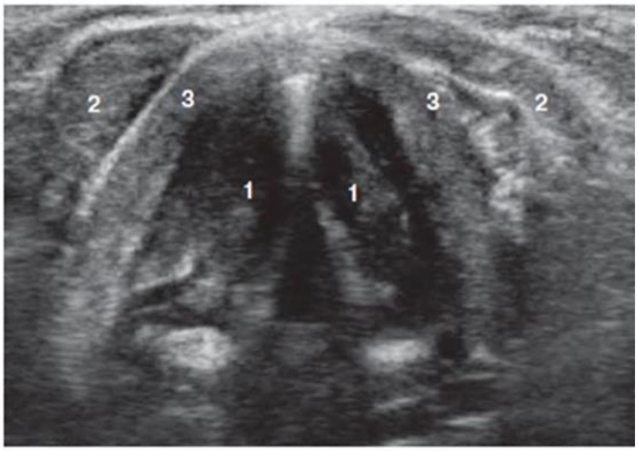


Figure 2. TLUS of the vocal cords in the open (quiet) position in the B-scan. (1) The true vocal cords, (2) short muscles of the neck, and (3) thyroid cartilage

*The patient is placed in a flat position with his neck slightly extended of the neck during TLUS. The ultrasound transducer is placed transversely over the anterior aspect of the middle portion of the thyroid cartilage. (Figure 1) False cords and true cords were identified using sonographic landmarks whenever possible. The true VCs are the hypoechogenic structures projected under the thyroid cartilage (Figure 2). Unilateral palsy in B-scan could be visualized as immobility of the VC during phonation or Valsalva maneuver (Figure 3).*

*According to Knyazeva et al., patients who are female and under 50 years old are more likely to have their vocal cords visualized by TLUS (89% of patients visualize their vocal cords). [1] According to structural features (a more acute angle of the thyroid cartilage) and laryngeal calcification, the approach does not appear accurate for male patients. It has been suggested that TLUS is a commonly used, non-invasive treatment. [1] It is convenient for the patient and does not increase the financial cost of preoperative workup because the thyroid surgeon himself can perform it. Furthermore, the mean length of the ultrasound*

*examination time is significantly shorter than the time spent performing flexible laryngoscopy (1.8 ± 0.86 min vs. 3.4 ±1.06 min; p < 0.0001) as reported by Masood et al. [2] TLUS is an effective method for function control of the vocal cords in most patients undergoing thyroid and parathyroid surgery. The vast majority of surgery candidates, who are typically female and young, might benefit from using this visualizing technique. Laryngeal calcifications are a limiting concern, necessitating the use of the DFL in 20% of cases, particularly in male patients. [1]*

*1] Knyazeva, Polina, et al. "Transcutaneous laryngeal ultrasonography (TLUS) as an alternative to direct flexible laryngoscopy (DFL) in the perioperative evaluation of the vocal cord mobility in thyroid surgery." Langenbeck's Archives of Surgery 403.8 (2018): 1015-1020.*

*2] Masood MM, Huang B, Goins A, Hackman TG (2018) Anatomicfactors affecting the use of ultrasound to predict vocal fold motion:a pilot study. Am J Otolaryngol 39(4):413–417. https://doi.org/10.1016/j.amjoto.2018.04.005*

3) When should a patient after a Covid-19 infection be operated on? What do the authors recommend in case of an infection although vaccinated?

*According the Association of Anaesthetics, the recommendation for patients with elective surgery should be individualised risk assessment and be planned within seven weeks of SARS-CoV-2 infection. [1] If surgery is considered the risks and benefits should be discussed:*

*a) The risk of mortality calculated using a validated risk score*

*b) Risk modifiers based on patient factors, SARS- CoV-2 infection (timing, symptoms) and surgical factors (risk of disease, complexity of surgery)*

*Within ten days of a diagnosis of SARS-CoV2 infection, patients should not undergo elective surgery because they may be contagious and pose a risk to the staff, and other patients.*

*Even after 7 weeks, patients with ongoing symptoms and those with moderate to severe COVID19 (such as those who were hospitalised) are still likely to be at higher risk of morbidity and mortality. As a result, postponing surgery, weighing this risk against any risks connected to the delay.*

*Whenever possible, preoperative COVID-19 vaccination should have been given to surgical patients in three doses, with the last dose given at least two weeks before the surgery.*

*1] El-Boghdadly, K., et al. "Timing of elective surgery and risk assessment after SARS-CoV-2 infection: an update: A multidisciplinary consensus statement on behalf of the Association of Anaesthetists, Centre for Perioperative Care, Federation of Surgical Specialty Associations, Royal College of Anaesthetists, Royal College of Surgeons of England." Anaesthesia 77.5 (2022): 580-587.*

4) Are there data that the material obtained by FNA is contaminated by the Sars-Cov2-Virus? If so, please describe.

*I searched pubmed, google scholar and scopus and i can't find data about SARS- CoV2 contaminated material.*

5) What is the relevance of preoperative ultrasound? I miss data about that, in particular with respect to TRAS-classification: Please describe.

*Ultrasound of the thyroid gland is a non-invasive examination and the first step in evaluating the thyroid parenchyma and its nodules [1]. The non-invasive nature of this examination makes it easy and quick to use, with low cost, but it is an operator-dependent examination. The thyroid nodule grading system that has been proposed is TIRADS. There are the following nodule classification systems, EU-TIRADS, K-TIRADS, ACR-TIRADS and BTA-TIRADS. EU TIRADS had the highest sensitivity (82.7%); ATA the highest specificity (66.4%). The study by Dutta et al. showed that FNA and the TIRADS system showed that both systems have a sensitivity of 80%. Ultrasound findings show microcalcifications have a sensitivity of 80% and a specificity of 86%. Also, the irregular shape and the taller than wider parameter have a sensitivity of 89% and 92%, respectively (2). During the pandemic, less contact with the patient is the first choice of the doctor and the health systems. The development of remote ultrasound is a safe option, although it is still at an early stage. The study by Jiang W et al,, on 22 patients with COVID-19 infection showed that it is possible to remotely perform an ultrasound examination using robotic arms and the 5g network (3).*

*1] Hauger B.R., Alexander E.K., Bible K.C., Doherty G.M., Mandel S.J., Nikiforov Y.E., Pacini F., Randolph G.W., Sawka A.M., Schlumberger M., et al. 2015 American thyroid association management guidelines for adult patients with thyroid nodules and differentiated thyroid cancer: The American thyroid association guidelines task force on thyroid nodules and differentiated thyroid cancer. Thyroid. 2016;26:1–33.*

*2] De D, Dutta S, Tarafdar S, Kar SS, Das U, Basu K, Mukhopadhyay P, Ghosh S. Comparison between Sonographic Features and Fine Needle Aspiration Cytology with Histopathology in the Diagnosis of Solitary Thyroid Nodule. Indian J Endocrinol Metab. 2020 Jul-Aug;24(4):349-354.*

*3] Jiang W, Zhao X, Gan T, Liu Y, Luo S, Zhu M, Chen S, Jiang Y, Wu M. Application of Tele-Ultrasound Robot During COVID-19 Pandemic: A Feasibility Study. J Ultrasound Med. 2022 Jun..*

**Response to reviewer 2**

1) I recommend a clear conclusion, that the role of the pandemic is multifactorial (disease itsself, social distancing, and other resulting effects - it is not proved which effects are the most)

*Studies and systematic reviews should follow accepted methodological guidelines during public health emergencies to give patients, doctors, and decision-makers reliable information. The disease itself, social distancing, and the devastating economic impact are just a few of the factors that play a significant part in the pandemic.*

## Comments by the Editor-in-Chief to Revised Submission

All reviewer comments were addressed adequately and the manuscript should be published in its present stage.
